# Peripherally misfolded proteins exacerbate ischemic stroke-induced neuroinflammation and brain injury

**DOI:** 10.1186/s12974-021-02081-7

**Published:** 2021-01-20

**Authors:** Yanying Liu, Kalpana Subedi, Aravind Baride, Svetlana Romanova, Eduardo Callegari, Christa C. Huber, Xuejun Wang, Hongmin Wang

**Affiliations:** 1 Division of Basic Biomedical Sciences and Center for Brain and Behavior Research, Sanford School of Medicine, University of South Dakota, Vermillion, SD 57069 USA; 2 Department of Chemistry, University of South Dakota, Vermillion, SD 57069 USA; 3 Department of Pharmaceutical Sciences, College of Pharmacy, University of Nebraska Medical Center, Omaha, NE 68106 USA

**Keywords:** Heart, Peripheral, Brain, Crosstalk, Ischemia, Stroke, Neuroinflammation, Protein aggregation, Exosome

## Abstract

**Background:**

Protein aggregates can be found in peripheral organs, such as the heart, kidney, and pancreas, but little is known about the impact of peripherally misfolded proteins on neuroinflammation and brain functional recovery following ischemic stroke.

**Methods:**

Here, we studied the ischemia/reperfusion (I/R) induced brain injury in mice with cardiomyocyte-restricted overexpression of a missense (R120G) mutant small heat shock protein, αB-crystallin (CryAB^R120G^), by examining neuroinflammation and brain functional recovery following I/R in comparison to their non-transgenic (Ntg) littermates. To understand how peripherally misfolded proteins influence brain functionality, exosomes were isolated from CryAB^R120G^ and Ntg mouse blood and were used to treat wild-type (WT) mice and primary cortical neuron-glia mix cultures. Additionally, isolated protein aggregates from the brain following I/R were isolated and subjected to mass-spectrometric analysis to assess whether the aggregates contained the mutant protein, CryAB^R120G^. To determine whether the CryAB^R120G^ misfolding can self-propagate, a misfolded protein seeding assay was performed in cell cultures.

**Results:**

Our results showed that CryAB^R120G^ mice exhibited dramatically increased infarct volume, delayed brain functional recovery, and enhanced neuroinflammation and protein aggregation in the brain following I/R when compared to the Ntg mice. Intriguingly, mass-spectrometric analysis of the protein aggregates isolated from CryAB^R120G^ mouse brains confirmed presence of the mutant CryAB^R120G^ protein in the brain. Importantly, intravenous administration of WT mice with the exosomes isolated from CryAB^R120G^ mouse blood exacerbated I/R-induced cerebral injury in WT mice. Moreover, incubation of the CryAB^R120G^ mouse exosomes with primary neuronal cultures induced pronounced protein aggregation. Transduction of CryAB^R120G^ aggregate seeds into cell cultures caused normal CryAB proteins to undergo dramatic aggregation and form large aggregates, suggesting self-propagation of CryAB^R120G^ misfolding in cells.

**Conclusions:**

These results suggest that peripherally misfolded proteins in the heart remotely enhance neuroinflammation and exacerbate brain injury following I/R likely through exosomes, which may represent an underappreciated mechanism underlying heart-brain crosstalk.

**Supplementary Information:**

The online version contains supplementary material available at 10.1186/s12974-021-02081-7.

## Background

Maintaining the integrity of the proteome is essential for cell survival and normal function; however, proteins frequently misfold due to genetic mutations, stress conditions, or unique metabolic challenge conditions [1]. Mammalian cells evolve three major mechanisms to maintain protein homeostasis, or proteostasis: molecular chaperones, the ubiquitin-proteasome system and the autophagy lysosome system [2]. These cellular surveillance mechanisms detect and repair misfolded proteins or, in many situations, completely annihilate the misfolded proteins from inside of cells. Conversely, impaired protein quality control causes proteotoxicity that has been linked to numerous devastating human diseases known as protein conformational disorders, including many neurodegenerative diseases, metabolic disorders, cardiomyopathies, liver diseases, and systemic amyloidosis [1].

Stroke is the fifth most common cause of death and is a leading cause of serious long-term disability in the USA. Ischemic stroke is caused by blockage of cerebral artery and is associated with neuronal loss and dysfunction. Following cerebral ischemia and reperfusion (I/R), mitochondria dysfunction, glutamate-induced excitotoxicity, and neuroinflammation occur, which leads to oxidative stress, protein damage, and aggregation [3, 4]. Proteostasis has been shown to play an important role in neuronal injury and functional recovery following I/R [5, 6].

Unlike stroke, many other neurological diseases involve specific misfolded pathogenic proteins that aggregate into seeds that serve as self-propagation agents for diseases’ progression [7]. This prion-like property of pathogenic proteins is seen in the pathology of Lewy body [8] and α-synuclein [9], tau [10], β-amyloid [11], mutant huntingtin [12], and various other neurodegenerative disease-associated proteins [13]. These studies focused on cell-to-cell pathological propagation between grafted cells and host cells in the brain. However, protein aggregates are also seen in peripheral organs such as the heart [14], kidney [15], and pancreas [16]. It remains unknown whether prion-like phenomenon can be propagated from peripheral tissues to the brain, leading to functional or pathological alterations in the brain or exacerbation of I/R induced brain injury. Importantly, the determinants affecting ischemic stroke pathophysiology and recovery are less understood. To address these questions, we here investigated the brain of a transgenic mouse expressing a missense (R120G) mutant alphaB-crystallin (CryAB^R120G^) selectively in the cardiomyocytes, which causes desmin-related cardiomyopathy [14, 17]. CryAB, also known as HSPB5, is a small heat shock protein expressed at high levels in the lens but is also ubiquitously expressed in other tissues such as the heart and skeletal muscles [18]. A majority of this line of CryAB^R120G^ mutant mice died of congestive heart failure between 6 and 7 months (m) of age with the pronounced accumulation of desmin and CryAB aggregates and impaired proteasome function in the heart, indicating impaired proteostasis in the cardiomyocytes [14]. This allows us to assess whether the animal at 2–3 months of age, when the cerebral blood flow is at normal levels, exhibits any functional or pathological alterations in the brain following I/R insult.

## Materials and methods

### Mice

Adult C57BL/6J wild-type (WT) mice (8–12 weeks of age; mean body weight, 25 g) were purchased from the Jackson Laboratories. CryAB^R120G^ mice and their non-transgenic (Ntg) littermate mice have been previously described [14]. All mice were housed in 3–4 per cage on a 14-h light/10 h dark cycle and were given free access to food and water. All animal procedures were approved by the Institutional Animal Care and Use Committee at the University of South Dakota and were in accordance with the National Institute of Health Guide for the Care and Use of Laboratory Animals.

### Transient middle cerebral artery occlusion

The CryAB^R120G^, Ntg (the CryAB^R120G^ WT littermates), or WT mice animals (male, at 2–3 m) were anesthetized with isoflurane and subjected either to middle cerebral artery occlusion (MCAO) or sham operation. Transient MCAO was induced by transient occlusion of the MCA as previously described [5, 19] using a modified intraluminal filament (Doccol, #7020910PK5Re, 7023910PK5Re). Briefly, male mice were anesthetized with 2% isoflurane and their body temperature was maintained at 37.0 ± 0.5 °C with an electronic thermostat-controlled warming blanket (Stoelting). Following permanent ligation of the external carotid artery (ECA), the filament was inserted into the ECA and guided toward the internal carotid artery through the common carotid artery to block the MCA. After 45 min (for the CryAB^R120G^ and Ntg mice) or 1 h (for C57BL/6J WT mice) of MCAO, the occluding filament was withdrawn to allow blood reperfusion. The blood flow of mice was monitored by a Doppler blood flowmeter (Vasamed). After animals were returned to their home cages, they were monitored closely over the next 4 h and then daily for the rest of the study.

### Measurement of infarct volumes

Following 24 h after I/R, mice brains were rapidly removed and stored at − 20 °C for 15 min to harden the tissue. The brains were sliced into 2-mm-thick coronal sections and then incubated with 2% 3,5-triphenyltetrazolium chloride (TTC, Sigma, #T8877) to evaluate the infarct volume as described previously [20, 21]. The infarct volume was manually analyzed using the ImageJ software (NIH).

### Assessment of neurological deficit scores after MCAO

The functional recovery test was performed at 1, 3, 5, and 7 days following MCAO, using a modified neurological severity score (mNSS) system that contains a battery of motor, sensory, reflex, and balance tests [22]. The mNSS was graded on a scale of 0 to 18 (normal neurological behavior score, 0; maximal neurological deficit, 18): 13–18, severe injury; 7–12, moderate injury; 1–6 mild injury [22].

### Radial arm water maze

The radial arm water maze (RAWM) was performed to assess animals’ learning and memory capability according to a previously described protocol [23]. Briefly, each mouse was gently placed into an arm and allowed to find the platform located in the goal arm. On day 1, mice were trained for 15 trials alternating the visible and hidden platform until the 12^th^ trial, and the final 3 trials were performed using the hidden platform. On day 2, all 15 trials were performed with the hidden platform. The number of incorrect arm entries (errors) was counted until mice found the goal arm or 60 s had passed. The experimenter was blind to the genotypes of mice at the time of testing.

### Y-maze analysis

The Y-maze analysis was performed to evaluate learning and memory as described previously [23]. Briefly, each mouse could move freely in start and the other arm of the Y-maze for 5 min (training phase). After 1 h, each mouse was placed in the start arm and allowed to explore all three arms (testing phase). Then, the number of entries to each arm was recorded for 2 min. An arm’s entry was counted when a mouse placed all four paws/legs inside an arm.

### Object recognition test

Mice were tested in a square wooden box (50 × 50 × 30 cm) and habituated to the empty box for 5 min the day before the first day of testing. On the first day of the test, mice were presented with two similar objects in the box and permitted to explore freely for 10 min. On the second day of the test, one of the two familiar objects was randomly replaced by a new object and mice were placed back into the box and allowed to explore the objects for 10 min. The amount of time spent exploring each object was recorded, and the relative exploration of the novel object was presented by a discrimination index (DI = (*T*_novel_ - *T*_familiar_)/(*T*_novel_ + *T*_familar_).

### String agility test

This test was used to test animals’ agility and grip capacity [23]*.* Briefly, each mouse was allowed to grasp a suspended string only using their forepaws and subsequently released by the experimenter. The maximum trial length was 60 s. The scoring system to assess the grasping capacity involved a five-scale system: 0-unable to remain on the string; 1-hangs by two forepaws; 2-attempts to climb to the string; 3-hangs by two forepaws and one or two hind paws around the string; 4-four paws and tail around the string; 5-escapes.

### Rotarod test

This was used to evaluate mouse balance and general motor function [23]. Each animal was placed on an accelerating Rotarod (Med Associates) and trained for three successive days with 3 trials each day. The maximum trial length was 250 s. The 4th day was the test day, with 3 trials for each mouse.

### Thioflavin S staining

Thioflavin S staining of protein aggregates in the brain was performed using a previously described method [23]. Brain sections were incubated with 70% ethanol (Fisher, #A40520) for 1 min and then 80% ethanol for 1 min. Slides were then incubated with 0.1% Thioflavin S (Sigma, #T1892) in 80% ethanol for 15 min. Slides were then rinsed with 80% ethanol for 1 min, 70% ethanol for 1 min, and twice with distilled water. Slides were mounted with Cytoseal 60 Medium (Richard-Allan Scientific, #8310-16), and images were acquired using a fluorescence microscope equipped with the ZEN 2.5 software (Carl Zeiss, Jena, Germany).

### Nissl staining

Brain sections that were rehydrated through 100% and 95 % alcohol to distilled water were stained in 0.1% Cresyl Violet (Acros Organics, #10510-54-0) solution for 5–10 min and then rinsed quickly in distilled water before were differentiated in 95% ethanol for 20 min and dehydrated in 100% ethanol twice for 5 min each. Finally, the sections were cleared twice in Xylene for 5 min and were mounted with Cytoseal 60 Medium (Richard-Allan Scientific, #8310-16). Images were captured under a microscope (Olympus), and 12–15 sections per animal were imaged. The number of surviving neurons in each section was quantified using the ImageJ software (NIH).

### Immunofluorescent staining

At 2 or 7 days post-MCAO, the PBS-perfused and paraformaldehyde-fixed brains were coronally sliced into 10 μm thickness sections on a Cryostat (Leica, Buffalo Grove, IL, USA) at − 20 °C. The brain sections or cell cultures (see below) were stained according to a previously described protocol [24]. Briefly, sections or fixed cell cultures were blocked with 5% bovine serum albumin (Fisher, #BP9706-100), and then incubated with anti-GFAP (1:1000, EMD Millipore, #AB5804; Lot 2013929), anti-Iba1 antibody (1:1000, FUJIFILM Wako, #019-19741; Lot LKF6437), anti-NeuN antibody (1:1000, Millipore, #ABN78, Lot 1979271), anti-CryAB antibody (1:100, Abcam, #ab13496), anti-TNFα antibody (1:100, Abcam, #ab1793, Lot GR3261538-1), anti-Myc antibody (Cell Signaling Technology, #2276, Lot 24), or anti-ubiquitin antibody (1:500, Abcam, #ab7780, Lot GR3196708-1). This was followed by incubation with Cy3- or Cy2-conjugated goat anti-rabbit or goat anti-mouse secondary antibodies (Jackson ImmunoResearch, #711-165-152) or Dylight 488 conjugated IgG, #35552, Lot QK224422). In some experiments, nuclei were counter-stained with a DNA binding dye, Hoechst 33342 (1:1000, ThermoFisher, #H3570). Images were captured with a fluorescence microscope equipped with the ZEN 2.5 software (Carl Zeiss). Then, 10–16 sections per animal in specific regions were imaged and data were quantified by using the NIH ImageJ software (NIH) according to previously described methods [25, 26]. When analyzing Iba1, we first counted all the Iba1-positive cells in every image, and the number was subsequently divided by the total (both neuronal and glial) number of cells (Hoechst 33342-positive cells) in the same picture. For GFAP analysis, we measured the GFAP fluorescence intensity and then divided it by the total (both neuronal and glial) nuclear (Hoechst-stained) intensity of the image [27].

### Western blot

Electrophoresis, transfer, and Western blot analysis of proteins were performed according to previously described methods [28]. The primary antibodies used were anti-CryAB (1:1000, Abcam; #ab13496), anti-Argonaute 1 (Argo1) (1:1000, Cell Signaling Technology, #5053, Lot 1), anti-Tsg101 (1:1000, Santa Cruz Biotechnology, #sc7964, Lot F0217), anti-ubiquitin (Ub) (1:1000, Cell Signaling Technology, #3936, Lot 14), anti-IL-1β (1:1000, Cell Signaling Technology, #63124), anti-IL-6 (1:1000, Cell Signaling Technology, #12912), anti-TNFα (1:1000, Cell Signaling Technology, #11948), and anti-β-actin (1:1000, Santa Cruz Biotechnology, #sc-1616, Lot G1615). The secondary antibodies used were conjugated with the infrared dyes (1:5000, LI-COR, #926-32211, Lot C80118-05; #92668072, Lot C71204-03; #926-33214, Lot C80207-07). Protein band intensities were measured using an Odyssey scanner (LI-COR) or the UN-SCAN-IT gel6.1 software (Silk Scientific).

### Isolation of exosomes from mouse blood plasma

Whole blood was collected from the facial vein of mice. Isolation of exosomes was performed as described previously with minor modification [29]. Briefly, 0.8-μm-filtered blood plasma samples were diluted by two folds with PBS and then centrifuged at 13,200×*g* at 4 °C for 22 min to remove microvesicles. The supernatant was filtered twice through 0.22 μm filters. Exosomes were pelleted by ultracentrifugation at 120,000×*g* with an SW41Ti rotor (Beckman Coulter). Pellets were washed once with PBS, and protein concentration was determined by the NanoDrop™ 2000/2000c spectrophotometers (Thermo Fisher).

### Nanoparticle tracking analysis of exosomes and treating WT mice with the exosomes

Prior to nanoparticle tracking analysis (NTA), samples were diluted at 1:100 in PBS and 1 ml exosome solution was used for NTA analysis to assess the size and number of exosome, a type of extracellular vesicle, using the NanoSight NS300 system (Malvern Instruments).

To determine whether the isolated exosomes influence brain function in WT mice, WT C57BL/6J mice were intravenously injected with 0.8 mg/kg of exosomes daily and animal motor and cognitive behaviors were examined after 2–3 weeks following the treatment using the behavioral test methods mentioned above. To determine the effect of isolated exosomes on I/R induced brain injury, the exosomes (3.75 mg/kg) were injected into WT C57BL/6J male mice through tail vein after 1 h of MCAO, and following the treatment, the mice were either euthanized after 24 h to assess their brain infarct volume or allowed to survive for 7 days to evaluate their survival rate and neurological deficit scores using methods described above.

### Isolation of protein aggregates, scanning electron microscopy, and measurement of protein aggregate size

Isolation of Triton X-100 insoluble protein aggregates from Ntg or CryAB^R120G^ mouse brains was performed according to previously described methods [30, 31]. Examining isolated protein aggregates with scanning electron microscopy (SEM) was performed with the field emission SEM (SIGMA, Zeiss). The samples were prepared by drop-casting on Si wafer and sputter coated with Au for the imaging. Secondary electron images were acquired by scanning with 1.5–2.0 kV acceleration voltage. A total of 33–38 aggregates from 3 to 4 samples were measured using the ImageJ software.

### Mass spectrometric analysis of proteins

The in-solution and in-gel protein digestion and processing were performed according to our previously described methods [28, 32]. The digested peptides were separated using Ultimate 3000RS UHPLC in nanoflow configuration (Thermo Scientific, Bremen, Germany) coupled to QExactive Plus (Thermo Scientific, Bremen, Germany) Quadrupole Orbitrap through a nanoelectrospray ion source using Full MS followed by ddMS2 (DDA) mode during 60 min [28]. Mascot Distiller v2.6.2.0 in-house licensed (www.matrixscience.com) and Proteome Discoverer v2.1 (Thermo Scientific) were used to generate the peak list at the mascot generic format (mgf) to identify +1 or multiple charged precursor ions from the mass spectrometry data file. Parent mass (MS) and fragment mass (MS/MS) peak ranges were 250–1800 Da (resolution 70000) and 65–2000 Da (resolution 17500), respectively. Mascot server v2.5.1 (www.matrix-science.com, UK) in MS/MS ion search mode (local licenses) was applied to conduct peptide matches (peptide masses and sequence tags) and protein searches against sp|P23927-1| CRYAB_MOUSE Alpha-crystallin B chain [Mus musculus] (1 sequence, 175 residues) as a wild type and a CRYAB_MOUSE Alpha-crystallin B mutant in house customized (^120^R replaced by G) (1 sequence, 175 residues), respectively. The following parameters were set for the search: carbamidomethyl (C) on cysteine was fixed and variable modifications included asparagine and glutamine deamidation and methionine oxidation. Only two missed cleavages were allowed; monoisotopic masses were counted; the precursor peptide mass tolerance was set at 30 ppm; fragment mass tolerance was 0.1 Da, and the ion score or expected cut-off was set at 5. The MS/MS spectra were searched with MASCOT using a 95% confidence interval (C.I. %) threshold (*p* < 0.05), with which minimum score of 13 was used for peptide identification, indicating identity or extensive homology. Additionally, the error-tolerant mode was set up at Mascot search to corroborate potential peptides unidentified at the first search. Mouse heart tissue from CryAB^R120G^ mice and Ntg mouse brains 24 h following ischemic stroke were used as positive and negative controls, respectively.

### Primary neuronal culture, exosome treatment, and ATP measurement

Primary mouse cortical neurons were prepared from wild-type C57BL/6J mice, as previously described [28] without inhibition of glial cell growth to generate the mixed neuron-glia cultures. The cells were cultured in poly-ornithine-coated 12-well plates for 7 days, and then treated with exosomes (10 μg/ml) isolated either from Ntg or CryAB^R120G^ mouse blood. After 24 h, the exosome-treated neuron-glia cultures were stained for protein aggregates using the Proteostat Aggresome Detection Kit (Enzo Life Sciences, #ENZ-51035) following the manufacturer provided protocol. Three independent experiments were performed in this study and at least 150 cells were analyzed in each experiment. To test the effect of exosomes on cell viability, the treated cells were collected for ATP assay using an ATP Bioluminescence Assay Kit CLS II (Sigma, #11699695001) according to the protocol provided by the manufacturer.

### CryAB^R120G^ seeding assay

To determine the prion-like property of mutant CryAB^R120G^ protein, mouse striatal cells (Coriell) were cultured in 12-well plates in complete medium containing the Dulbecco’s modified Eagle medium supplemented with 10% fetal bovine serum and penicillin/streptomycin (Thermo Fisher). Cells were transfected with myc-flag-CryAB plasmid (OriGene, #MR201515) encoding the myc-flag-tagged mouse CryAB protein. After 24 h, the cells were transduced with 500 ng/ml of Triton X-100 insoluble aggregates isolated either from Ntg or CryAB^R120G^ mouse brains using Trans-Hi™ transfection reagent (FormuMax, #F90101TH). The transduced cells were fixed and subjected to immunocytochemical staining with an anti-Myc antibody (see above *Immunofluorescent staining* part) 24 h following the transduction.

### Statistical analysis

Statistical analyses were conducted using GraphPad Prism version 7.0 statistical software. Differences between two groups were assessed using unpaired *t* test. Significant differences between more than two groups were analyzed using one-way or two-way ANOVA followed by Tukey’s post hoc test or Sidak’s multiple comparisons test. *P* < 0.05 was regarded as statistically significant.

## Results

### Cardiomyocyte-restricted expression of misfolded CryAB^R120G^ exacerbated I/R-induced brain injury and cognitive and motor defects

**Fig. 1:**
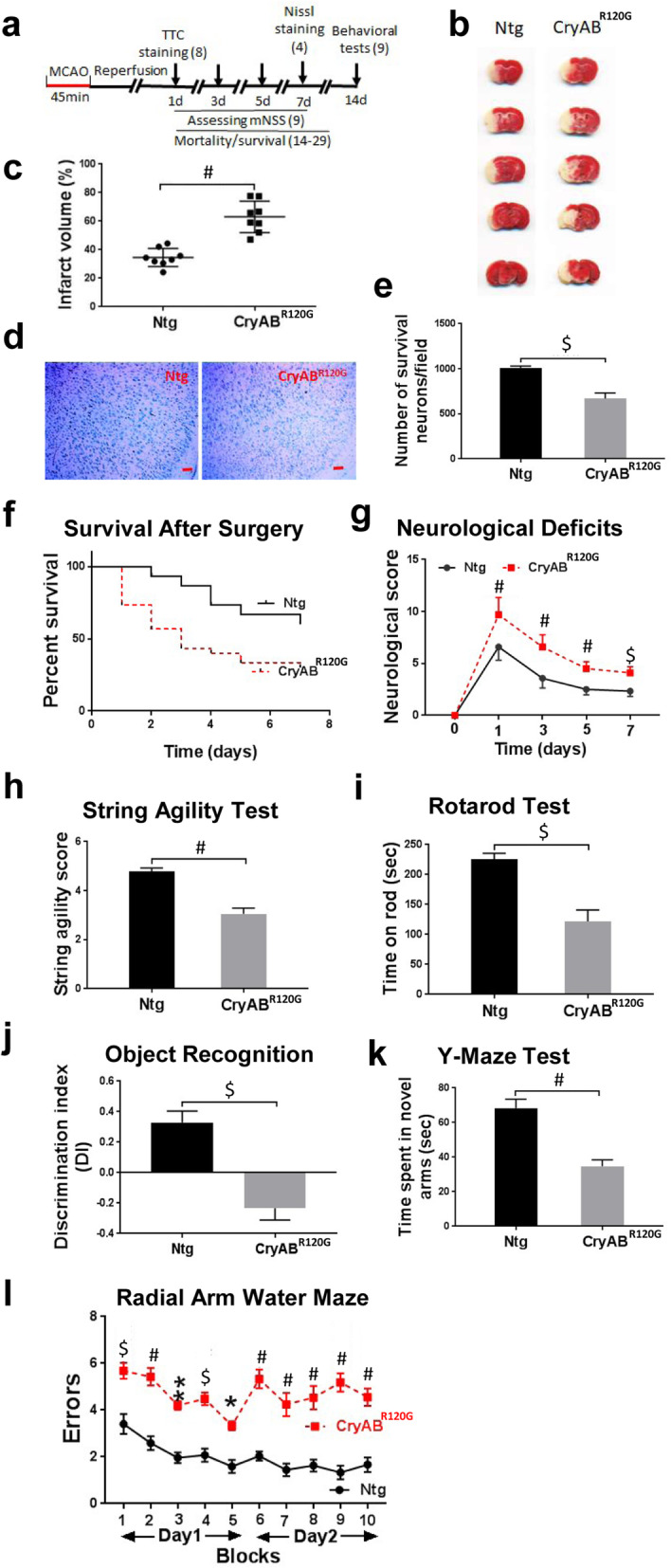
Cardiomyocyte-restricted expression of misfolded CryAB^R120G^ protein exacerbated I/R-induced brain injury and cognitive and motor deficits. **a** Diagram of the experimental design, the numbers in the parentheses indicating the number of animals used. **b** TTC staining mouse brains after 24 h of reperfusion. **c** Quantitative analysis of (**b**). **d** Representative images of Nissl staining results in the peri-infarct cortical area after 7 days of reperfusion. Scale bar, 100 μm. **e** Quantitative analysis of (**d**) from 4 mice with 12–15 sections/animal. **f** Survival rate of mice after MCAO. **g** Functional recovery of mice after MCAO. **h** String agility test results after 14 days of reperfusion. **i** Rotarod test results after 14 days of reperfusion. **j** Object recognition test results after 14 days of reperfusion. **k** Y-maze test results after 14 days of reperfusion. **l** Radial arm water maze (RAWM) results after 14 days of reperfusion. Numerical data are shown as mean ± SEM; *n* = 9 (the beginning numbers for the survival test was 14 for Ntg mice and 29 for CryAB ^R120G^ mice). *p < 0.05, **p < 0.01, $*p* < 0.001, #*p* < 0.0001

To determine whether peripherally impaired proteostasis influences brain injury and functional recovery, we challenged both CryAB^R120G^ male mice and their male Ntg littermates with I/R at 2–3 m. At this time, their cardiac outputs were still normal [14]; therefore, we examined brain injury and functional recovery. Cerebral blood flow did not differ between Ntg and CryAB^R120G^ mice before, during, or after MCAO (Supplementary Fig. S1). Following 45 min of MCAO and 24 h of reperfusion (Fig. 1a), CryAB^R120G^ mice showed increased infarct volume compared to their Ntg littermates (Fig. 1b, c). This result was further confirmed by the Nissl staining data, which showed fewer neurons surviving in CryAB^R120G^ mouse brains in comparison to Ntg mouse brains after 7 days of reperfusion (Fig. 1d, e). Consistently, CryAB^R120G^ mice also showed a higher mortality rate (Fig. 1f) and more severe neurological deficits as reflected by the mNSS (Fig. 1g) than their Ntg littermates. Prior to I/R insult, however, both CryAB^R120G^ mice and the Ntg littermates did not reveal any evident neurological deficits (mNSS = 0 for both types of animal). Using relatively sensitive methods, we observed slightly reduced or no altered motor and cognitive performance in the CryAB^R120G^ mice compared to the Ntg mice prior to I/R procedure (Supplementary Fig. S2). After 14 days of reperfusion, however, CryAB^R120G^ mice exhibited greater reduction in their motor function compared to Ntg mice in the string agility (Fig. 1h) and the rotarod tests (Fig. 1i). CryAB^R120G^ mice also showed poorer performance in the object recognition test (Fig. 1j), Y-maze test (Fig. 1k), and RAWM test (Fig. 1l) in comparison to their Ntg littermates, indicative of exacerbated cognitive dysfunctions in CryAB^R120G^ compared to Ntg mice. Thus, peripheral expression of misfolded CryAB^R120G^ protein aggravates I/R-induced brain injury and neurological deficits.

### Cardiomyocyte-restricted expression of misfolded CryAB^R120G^ exacerbated I/R induced neuroinflammation

**Fig. 2:**
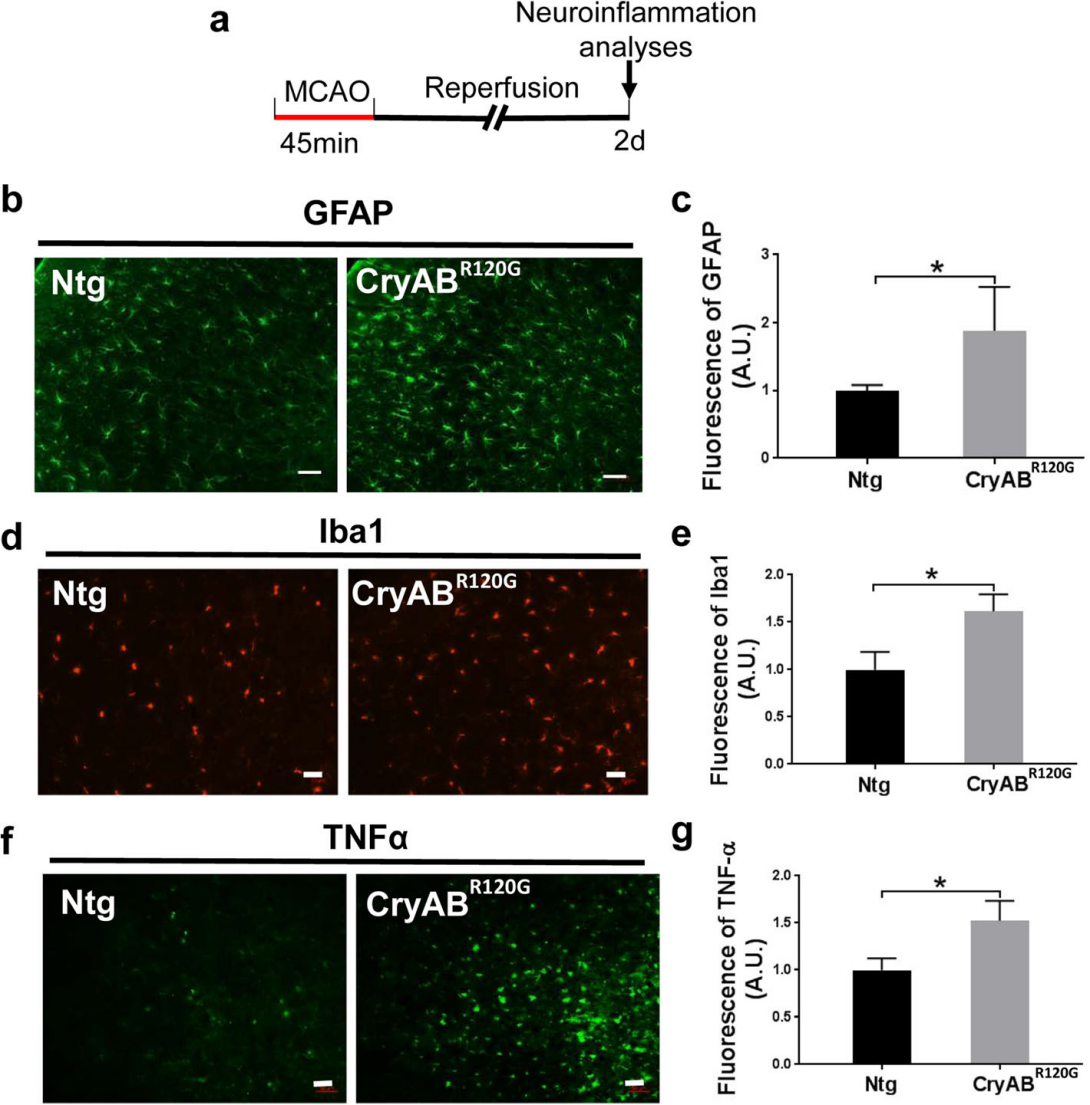
Cardiomyocyte-restricted expression of misfolded CryAB^R120G^ protein enhanced I/R-induced neuroinflammation in the brain. **a** Diagram of the experimental design. **b** Immunofluorescence staining of GFAP in the peri-infarct zone brain of mice after MCAO. Scale bar, 50 μm. **c** Quantitative analysis of (**b**). **d** Immunofluorescence staining of Iba1 in the brain of mice after MCAO. Scale bar, 50 μm. **e** Quantitative analysis of (**d**). **f** Immunofluorescence of TNFα in the cortical infarct brain of mice after MCAO. Scale bar, 50 μm. **g** Quantitative analysis of (**f**). Data are shown as mean ± SD; for immunostaining, 10–16 sections per animal were imaged and analyzed using the ImageJ software. *N* = 3–4. *p < 0.05, **p < 0.01

To determine whether cardiomyocyte-restricted expression of misfolded CryAB^R120G^ proteins alters I/R triggered neuroinflammation, immunohistochemistry was utilized to examine astrocytes and microglia in the mouse brains after 2 days of I/R (Fig. 2a). Both GFAP (glial fibrillary acidic protein, an astrocyte marker) (Fig. 2b, c) and Iba1 (a microglial marker) (Fig. 2d, e) showed a higher level of immunoreactivity in the CryAB^R120G^ mouse brains than in the Ntg brains. This is indicative of increased activation of astrocytes and microglia in CryAB^R120G^ mouse brain following I/R. Importantly, the immunoreactivity of TNFα, a potent activator of the immune system [33], astrocyte s[34], and microglia [35], was also higher in CryAB^R120G^ mouse brain than in Ntg mouse brain after 2 days of I/R (Fig. 2f, g), suggesting pronounced neuroinflammation occurring in CryAB^R120G^ mouse brains. To gain further insight into the inflammatory response at the early time points, we performed Western blot analysis of the pro-inflammatory molecules, IL-1β, IL-6, and TNFα at 0, 6, 12, and 24 h following I/R. We observed a dramatic increase of the three pro-inflammatory molecules selectively in CryAB^R120G^ mouse brain (Supplementary Fig. S3). Collectively, these data reveal that cardiomyocyte-restricted expression of misfolded CryAB^R120G^ protein enhances I/R induced neuroinflammation.

### Cardiomyocyte-restricted expression of misfolded CryAB^R120G^ protein enhanced I/R induced protein aggregation in the brain

**Fig. 3:**
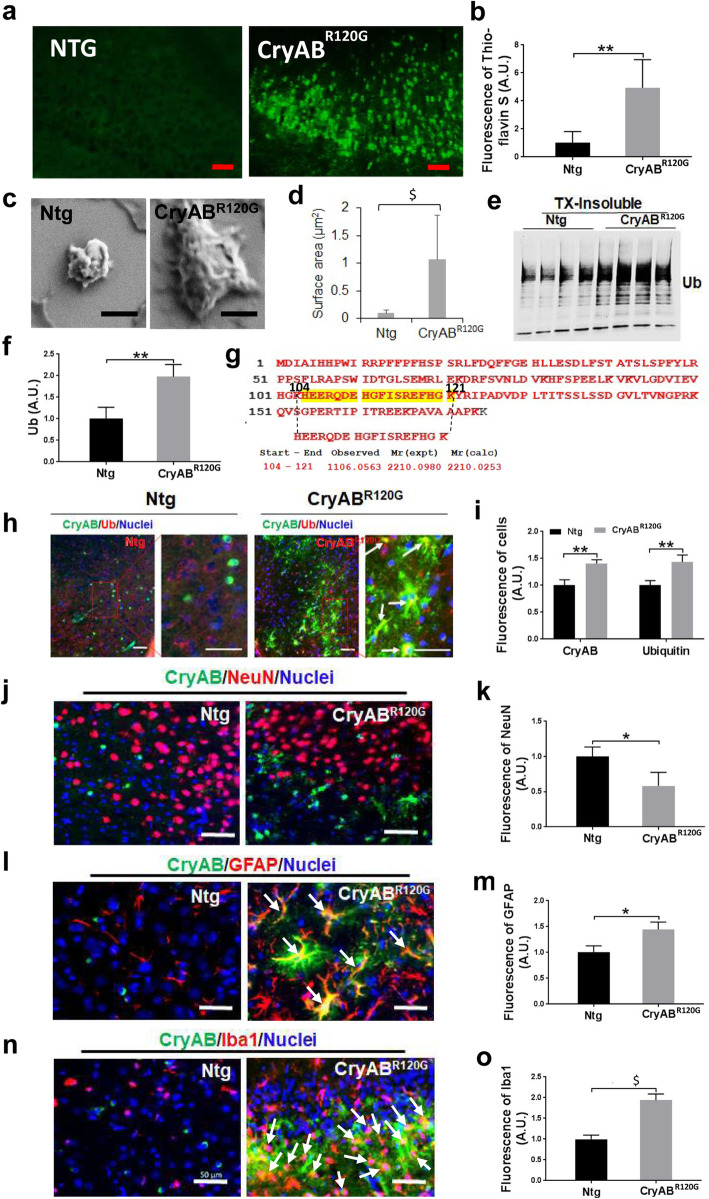
Cardiomyocyte-restricted expression of misfolded CryAB^R120G^ protein enhanced I/R-induced protein aggregation in the brain. **a** Thioflavin S staining of the cortical peri-infarct zone of mouse brain at day 7 following MCAO. Scale bar, 50 μm. **b** Quantitative analysis of (**a**). **c** Insoluble protein aggregates from mouse brains were analyzed with a scanning electron microscope. Scale bar, 500 nm. **d** Measured protein aggregate size. **e** Western blot analysis of ubiquitin (Ub) protein level from the Triton X100-insoluble CryAB proteins in the brain of mice after MCAO. **f** Quantitative analysis of (**e**). **g** Tandem mass spectrometric analysis of the protein aggregates isolated from the CryAB^R120G^ brains following I/R indicate that they contain the mutant CryAB^R120G^ protein. **h** Co-localization of (poly) ubiquitin and CryAB in the brain (peri-infarct, cortex) of mice after MCAO. Scale bar, 50 μm. **i** Quantitative analysis of (**h**). **j** Co-staining of NeuN and CryAB in the brain of mice after MCAO. Scale bar, 50 μm. **k** Quantitative analysis of (**j**). **l** Co-staining of GFAP and CryAB in the brain of mice after MCAO. Arrows, showing colocalization of GFAP with CryAB as indicated by yellow color. Scale bar, 50 μm. **m** Quantitative analysis of (**l**). **n** Co-staining of Iba1and CryAB in the brain of mice after MCAO. Arrows, showing colocalization of Iba1 with CryAB as indicated by yellow in color. Scale bar, 50 μm. **o** Quantitative analysis of (**n**). For immunostaining, 10–15 sections per animal were imaged and analyzed with the ImageJ software. Numerical data are shown as mean ± SD; *n* = 3 or 4. * *p* < 0.05, **p < 0.01, $*p* < 0.001

As I/R impairs the proteasome and causes protein aggregation in the brain [3–5], we next determined whether cardiomyocyte-restricted expression of misfolded CryAB^R120G^ influences I/R induced protein aggregation in the brain. Accordingly, we performed Thioflavin-S staining on the Ntg and CryAB^R120G^ mouse brains before I/R, and 1 or 7 days after I/R. Before I/R, the CryAB^R120G^ mouse brain showed faint Thioflavin-S staining, whereas the Ntg mouse brain was absent of Thioflavin-S staining (Supplementary Fig. S4). Following 1 or 7 days of I/R, we observed much brighter fluorescence in the CryAB^R120G^ mouse brain than in the Ntg brain and these stained aggregates were seen at 24 h following I/R (Supplementary Fig. S5, Fig. 3a, b). We then isolated the Triton-X100 insoluble aggregates from the Ntg and CryAB^R120G^ brains following I/R and examined their morphology under an electron microscope. In support of Thioflavin-S staining results, protein aggregates isolated from the CryAB^R120G^ mouse brains were larger and showed more cluster-like features than those isolated from Ntg mouse brains (Fig. 3c, d). The majority of protein aggregates isolated from the CryAB^R120G^ mouse brains were either large (> 1 μm^2^) or medium sized (0.1–1 μm^2^) inclusions, whereas most of those isolated from Ntg mouse brains were small-sized (< 0.1 μm^2^) aggregates (Supplementary Fig. S6). Western blot analysis of the protein aggregates following sonication in an SDS-containing buffer demonstrated that the proteins were (poly)ubiquitinated with a higher level of (poly)ubiquitin in CryAB^R120G^ mouse brain than in Ntg brain (Fig. 3e, f). To further determine whether the CryAB^R120G^ mutant protein translocated from the heart to the brain to induce the normal CryAB becoming misfolded proteins, we isolated protein aggregates from the brain and then performed tandem mass spectrometry. The protein aggregates isolated from CryAB^R120^ mouse brains following ischemic stroke contained not only normal CryAB but also CryAB^R120G^ mutant protein (Fig. 3g). As expected, CryAB^R120G^ protein was also identified from the protein aggregates of CryAB^R120G^ mouse heart (Supplementary Fig. S7A), whereas protein aggregates isolated from the Ntg mouse brains following ischemic stroke only contained wild-type CryAB (Supplementary Fig. S7B). Immunohistochemical staining confirmed the colocalization of CryAB protein with (poly)ubiquitin protein in CryAB^R120G^ mouse brains, and both CryAB and (poly)ubiquitin fluorescence intensities were higher in CryAB^R120G^ mouse brains than in Ntg mouse brains at day 7 following I/R (Fig. 3h, i). NeuN immunoreactivity in CryAB^R120G^ mouse brains was reduced at day 7 after I/R (Fig. 3j, k), indicating neuronal loss. By contrast, GFAP (Fig. 3l, m) and Iba1 immunoreactivities (Fig. 3n, o) were dramatically increased in some regions, suggesting activation of astrocytes and microglia. Moreover, both GFAP and Iba1 showed a relatively high degree of colocalization with CryAB aggregates as indicated by the yellow color (Fig. 3l, n, pointed by arrows). Therefore, cardiomyocyte-restricted expression of misfolded CryAB^R120G^ protein facilitates I/R induced protein aggregation in the brain.

### CryAB^R120G^ mice-derived exosomes showed an elevated level of exosome marker and CryAB proteins

**Fig. 4:**
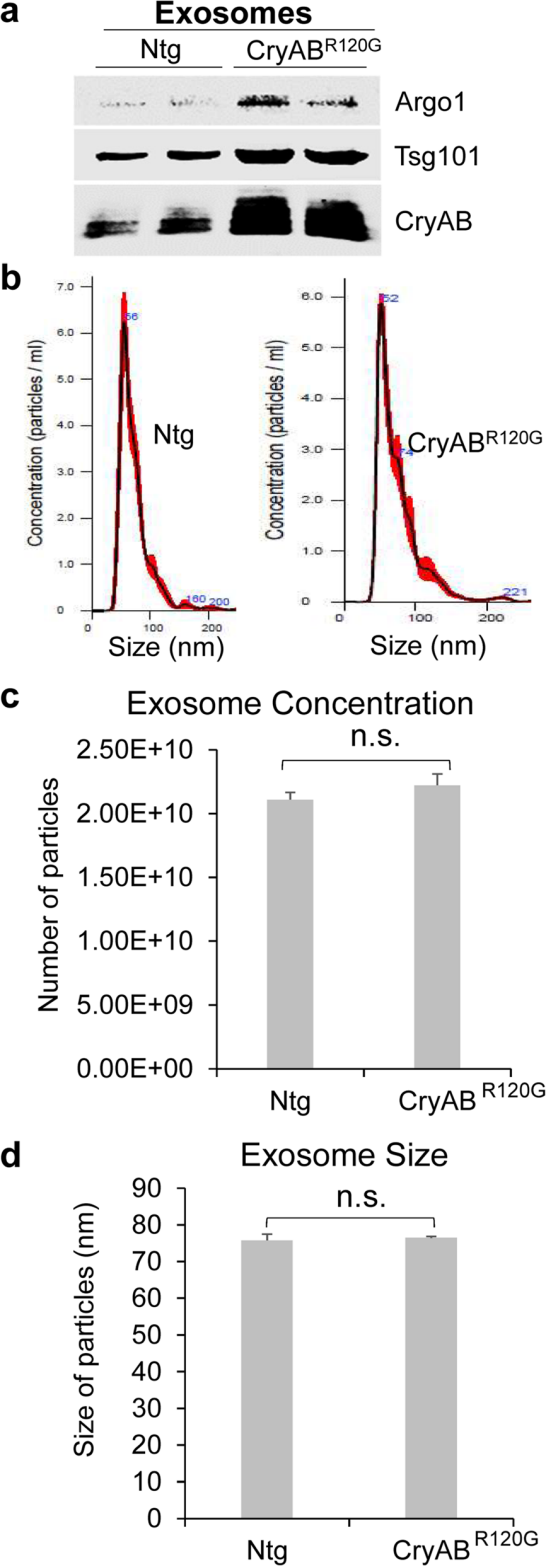
Isolation and characterization of CryAB^R120G^ mice-derived exosomes. **a** Exosomes isolated from the blood of CryAB^R120G^ mice expressed the exosome marker proteins such as Argonaute 1 (Argo1) and Tsg101. **b** NTA analysis of exosomes isolated from Ntg (left panel) or CryAB^R120G^ (right panel) mice. **c** The concentration of the CryAB^R120G^ and Ntg exosomes did not differ each other. **d** The size of the CryAB^R120G^ and Ntg exosomes did not differ each other. Data are shown as mean ± SD; *n* = 3

Exosomes, one type of extracellular vesicles that contain numerous proteins, lipids, RNAs, and other substances, play a crucial role in mediating cell-to-cell and heart-to-brain communications [36]. We next determined whether the increased protein aggregates seen in the CryAB^R120G^ mouse brains were mediated by circulating exosomes. Accordingly, we isolated exosomes from the non-stroke animal blood derived from either CryAB^R120G^ mice or their Ntg littermates and initially characterized them. Exosomes isolated from both CryAB^R120G^ mouse blood (hereafter referred to as CryAB^R120G^ exosomes) and Ntg mouse blood (referred to as Ntg exosomes) expressed exosomal markers, such as Argo1 and Tsg101, despite a remarkably higher level of these proteins in CryAB^R120G^ exosomes than in Ntg exosomes (Fig. 4a). Intriguingly, the level of CryAB protein was also dramatically higher in CryAB^R120G^ exosomes than in Ntg exosomes (Fig. 4a); however, the concentration (Fig. 4b, c) and size (Fig. 4b, d) of exosomes in the two types of mouse blood did not differ.

### CryAB^R120G^ mice-derived exosomes exacerbated I/R-induced brain injury and neurological deficits after administration to WT mice

**Fig. 5:**
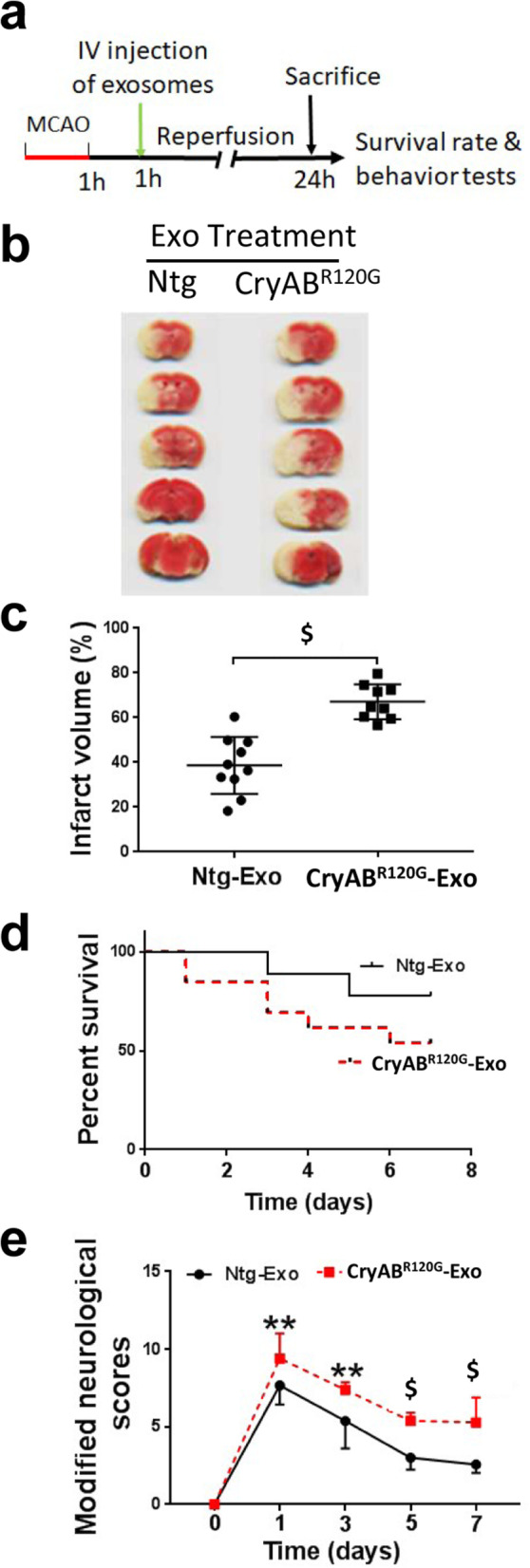
Treatment of WT mice with CryAB^R120G^ mice-derived exosomes worsened I/R-induced brain injury. **a** Diagrammatic illustration of the experimental design of treatment of WT mice with exosomes. **b** TTC staining of WT mouse brains treated with exosomes isolated from Ntg mice or CryAB^R120G^ mice. **c** Quantitative analysis of (**b**). **d** Survival rate of mice following MCAO and exosome treatment. **e** Neurological deficits following MCAO and exosome treatment. Data are shown as mean ± SD; *n* = 10 for Ntg-Exo treatment and *n* = 9 for CryAB-Exo treatment in (**c**–**e**). **p < 0.01, $*p* < 0.001

We examined whether CryAB^R120G^ and Ntg exosomes showed distinct effects on I/R induced brain injury and neurological deficits following I/R in WT mice. Toward this end, WT mice were subjected to 1 h MCAO and after 1 h of reperfusion, mice were intravenously treated with CryAB^R120G^ exosomes or Ntg exosomes before they were sacrificed at 24 h to assess neuronal injury or were allowed to survive for 7 days to evaluate their survival and functional recovery (Fig. 5a). The mice injected with CryAB^R120G^ exosomes showed increased infarct volume (Fig. 5b, c), reduced survival rate (Fig. 5d), and increased neurological deficits (mNSS, Fig. 5e) when compared with those injected with Ntg exosomes. To further examine whether injection of WT mice with the exosomes alters protein aggregation in the brain following I/R insult, we performed Thioflavin S staining of brain sections 24 h after injection. Injection of CryAB^R120G^ exosomes significantly increased I/R-induced protein aggregation when compared to the injection of Ntg exosomes (Supplementary Fig. S8). Hence, these data support that the exosomes, at least partially, mediate the transfer of the mutant CryAB^R120G^ proteotoxicity from the heart-to-brain, leading to increased brain injury and neurological deficits.

### The misfolded CryAB^R120G^ protein showed prion-like propagation when transduced into cultured cells

**Fig. 6:**
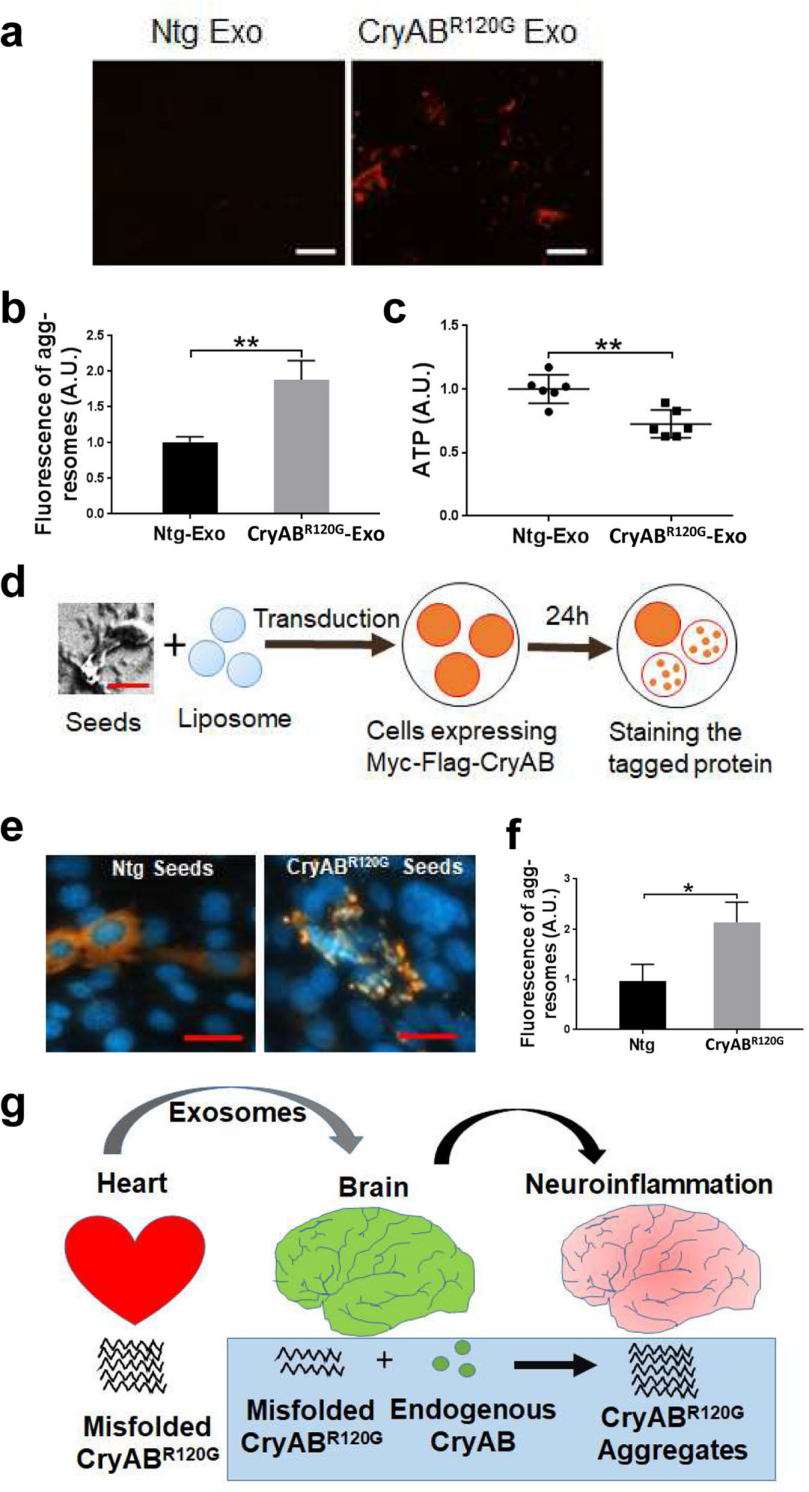
The Prion-like propagation of CryAB^R120G^ protein in cell cultures. **a** Protein aggregates in primary mixed neuron-glia cultures detected with a Proteostat aggresome detection kit following incubation with the exosomes (Exo) isolated from Ntg or CryAB^R120G^ mice. Scale bar, 50 μm. **b** Quantitative analysis of (**a**). **c** Cultured neurons treated with CryAB^R120G^ exosomes showed a reduced ATP level. **d** Schematic illustration of cell culture assay to determine the prion-like property of CryAB^R120G^ protein. **e** Immunocytochemical staining of transfected myc-flag-tagged wild-type mouse CyAB in neural cells following transduction of CryAB^R120G^ aggregate seeds. Scale bar, 20 μm. **f** Quantitative analysis of (**e**). **g** Hypothetical model explaining the prion-like propagation of misfolded CryAB^R120G^ protein from the heart to brain. Exosomes containing CryAB^R120G^ protein can be transported from the heart to brain through blood circulation. In the brain, CryAB^R120G^ protein propagates in a prion-like phenomenon, and causes increased neuroinflammation and abnormal brain function. Data are shown as mean ± SD; *n* = 6 for (**c**) and *n* = 3 for (**b**), (**f**). *p < 0.05, **p < 0.01

To further understand how the misfolded CryAB^R120G^ protein induces neuronal injury in the brain, we treated the primary mouse cortical neuron-glia mix cultures with the two types of exosomes. After 24 h following the incubation of exosomes with cultured cells, pronounced protein aggregation was observed selectively in the cells incubated with CryAB^R120G^ exosomes but not in those incubated with Ntg exosomes (Fig. 6a, b). CryAB^R120G^ exosome-induced protein aggregates were also associated with reduced ATP levels in the cultures (Fig. 6c), suggesting possible mitochondrial dysfunction occurred in the cells.

As mutant CryAB^R120G^ proteins show dramatic aggregation and form large inclusions in the heart [14], we determined whether misfolded CryAB^R120G^ translocated from the heart-to-brain showed the prion-like propagation property in cell cultures. Therefore, we isolated CryAB^R120G^ protein aggregates from either CryAB^R120G^ or Ntg mouse brains (Fig. 3c, d) and transduced them into cultured neural cells expressing the *myc*-tagged WT mouse CryAB. Then, the cells were stained for analyzing the *myc*-tagged CryAB protein aggregation status 24 h following the transduction (Fig. 6d). Transduction of the aggregate seeds into cells was performed by mixing isolated CryAB^R120G^ protein seeds with liposomes before treating cells, as previous data have shown that this would facilitate direct transduction of seeds into the cultured cells, thereby maximizing seed detection efficiency [37]. As expected, the cells receiving CryAB^R120G^ aggregate seeds exhibited remarkable CryAB aggregates (Fig. 6e, right panel), while those receiving Ntg protein aggregate seeds showed diffused CryAB staining (Fig. 6e, left panel). Moreover, the fluorescence intensity in cells transduced with CryAB protein aggregate seeds was significantly higher than those transduced with Ntg seeds (Fig. 6f). Taken together, these data strongly suggest that the mutant CryAB^R120G^ protein can translocate to the brain from the heart via exosomes or directly disrupting BBB function, where it induces prion-like propagation and thereby results in the formation of large CryAB aggregates, disruption of neuronal survival and function, and increased neuroinfammation (Fig. 6g).

## Discussion

Protein aggregation can be found in peripheral organs, such as the heart [14], kidney [15], and pancreas [16]. It remains unknown whether prion-like phenomenon can be propagated from peripheral tissues to the brain and whether protein aggregates in a peripheral organ influence the pathological outcome and functional recovery following I/R-induced brain injury. Here, we demonstrated that following acute I/R-induced brain injury, CryAB^R120G^ mice showed increased infarct volume and neurological deficits, enhanced neuroinflammation, and formation of large CryAB protein aggregates positive in ubiquitin staining. Moreover, we further demonstrated that WT mice intravenously injected with exosomes isolated from CryAB^R120G^ mouse blood showed similar phenotypes as CryAB^R120G^ mice following I/R. Addition of either the purified CryAB^R120G^ exosomes or protein aggregates into cell cultures induced pronounced protein aggregates, exhibiting self-propagation of the CryAB^R120G^ misfolding. Therefore, our data highlight the significance of peripheral proteostasis in influencing neuroinflammation, neuronal injury, and brain functional recovery.

The increased neuronal injury and neuroinflammation observed here were likely not caused by altered cardiac functions in the CryAB^R120G^ mice, as the mice still show normal cardiac output at 2–3 months of age [14] when they were used for the experiments. Given that the animal’s cardiac contractile function was not reduced at this stage [14], the observed neuropathological alterations and neurological deficits should not be caused by the insufficient cerebral blood supply. Moreover, the CryAB^R120G^ mice did not show cardiac inflammation, as their heart did not have fibrosis, a pathological condition frequently considered as a sign of cardiac inflammation [38] at 2–3 months of age [14]. Our data strongly support that misfolded CryAB^R120G^ proteins derived from the heart contribute, at least partially, to the aggravated brain injury and impaired brain functions. First, we observed heightened pro-inflammatory molecules and increased activation of microglia and astrocytes in CryAB^R120G^ mouse brains following I/R. Second, CryAB^R120G^ exosomes contained a high level of CryAB protein, presumably the CryAB^R120G^ protein. Finally, CryAB^R120G^ exosomes exacerbated I/R-inducted brain injury and neurological deficits. Hence, our results strongly support the role of heart-to-brain translocation of CryAB^R120G^ proteins in triggering increased neuroinflammation and brain dysfunction following I/R. On the other hand, I/R may also further enhance the production of the CryAB^R120G^ exosomes containing increased misfolded proteins. Since exosomes can selectively reach the lesion areas of the brain in neurological disease mice [39], this interplay could exacerbate brain injury following I/R.

One interesting phenomenon we observed here was that CryAB^R120G^ exosomes contained higher levels of the exosomal marker proteins than Ntg exosomes; however, the two types of exosomes did not differ in concentration and size. The elevated exosome marker proteins may be required for the targeting of misfolded CryAB^R120G^ protein to the exosome to facilitate the formation of exosomes. It is also possible that the increased exosome marker proteins are required for potentiating the spreading of the misfolded protein in the pathological condition of misfolded proteins. Our results are in accordance with previous studies showing that the misfolded protein is associated with increased exosomal marker proteins [40].

The misfolded CryAB^R120G^ protein enters the brain, likely using two pathways, by exosomes and through disrupting BBB. There is compelling evidence supporting that exosomes released by cells in the periphery can pass the BBB and deliver the cargos to brain cells particularly under inflammatory conditions [41]. Alternatively, the mutant CryAB protein may first enter the endothelial cells of the brain-blood vessels to disrupt their proteostasis and impair the BBB integrity before directly crossing BBB. Once inside brain cells, mutant CryAB^R120G^ proteins undergo the prion-like propagation of protein misfolding, recruiting endogenous normal CryAB protein in brain cells to induce CryAB aggregation (Fig. 6g). However, we observed much weaker Thioflavin S labeled protein aggregation in the WT brains (Fig. S7) of the mice injected with CryAB^R120G^ exosome than CryAB^R120G^ transgenic mouse brains (Fig. 3a). This may suggest that the injected CryAB^R120G^ exosome dose was lower than the circulating CryAB^R120G^ exosomes found in the transgenic mouse blood. The pronounced protein aggregates seen in the primary cortical neuron-glia mix cultures following incubation with CryAB^R120G^ exosomes (Fig. 6a, b) strongly suggest this possibility existing. As the endogenous CryAB itself is a small heat shock protein and molecular chaperone, it binds mutant CryAB^R120G^ proteins to prevent protein aggregation and cell death [42, 43]. Indeed, augmentation of intact CryAB consistently confers neuroprotection against ischemia-induced neuronal injury [44, 45], while CryAB deficiency leads to increased lesion size and diminished neurologic function after stroke [44]. In the condition of ischemic stroke, however, the amount of chaperone proteins is insufficient to deal with overwhelming proteotoxic stress [46, 47]. Consequently, the mutant CryAB^R120G^ proteins derived from the heart can become overwhelming to potentiate protein aggregation, neuroinflammation, and neuronal injury.

Although the astrocytes of mouse brains contain more CryAB than neurons [48, 49], we found that increased CryAB immunoactivity was predominantly located in both microglia and neurons in CryAB^R120G^ mouse brains but not in astrocytes in the absence of any insults (data not shown). Following I/R, however, remarkably increased CryAB immunoactivity was seen mainly in astrocytes and microglia but not in neurons. This phenomenon may result from selective loss of the neurons following the uptake of CryAB^R120G^ protein, due to the nature of its toxicity, especially in the context of I/R. Thus, the heart-derived CryAB^R120G^ protein induces neuronal damage and glial activation in a prion-like dependent manner. Following I/R and neuronal death, the debris including CryAB^R120G^ aggregates might be further ingested by astrocytes and microglia to induce activation of additional glial cells, causing neuroinflammation in glial cells and brain dysfunction (Fig. 6g). Increased levels of glial immunofluorescence reactivity, pro-inflammatory cytokines, and colocalization of CryAB^R120G^ aggregates with the astrocyte marker, GFAP, and microglial marker, Iba1, strongly support this possibility.

It is possible that CryAB aggregates activate NLRP3 or NLRP3–ASC inflammasome [NACHT, LRR, and PYD domains-containing protein 3 (NLRP3)-apoptosis-associated speck-like protein containing a CARD (ASC)], an important sensor of innate immunity, in glial cells, just like β-amyloid and tau aggregates in Alzheimer’s disease [50–52] and amyotrophic lateral sclerosis (ALS) protein in ALS [53]. Many cytokines derived from the reactive astrocytes and microglia are deleterious to neuronal survival [54] and can exacerbate brain injury following I/R. Moreover, CryAB aggregates in glial cells can directly impair production of the neurotrophic factors and protection from glia [55], leading to enhanced neuronal injury following I/R.

Stroke occurs in isolation (no other co-occurring conditions) in only 6% of patients, and most of the stroke patients have comorbidity conditions [56]. Impaired proteostasis is frequently seen in peripheral organs in the elderly [57, 58], but very little is known about the impact of peripherally impaired proteostasis on ischemic stroke-induced brain injury. Following I/R, we observed a great impact of CryAB^R120G^ proteins on brain dysfunction and pathological alterations, including increased infarct size and neurological deficits, as well as pronounced neuroinflammation and dramatic accumulation of large protein aggregates. Although the exact mechanism underlying the exacerbation of I/R-induced brain injury by cardiomyocyte-derived CryAB^R120G^ proteins remains to be explored, the translocation of CryAB^R120G^ from the heart to brain and self-propagation of CryAB^R120G^ misfolding in the brain may play an important role in worsening I/R brain injury. When the isolated CryAB^R120G^ exosomes were added into primary mixed neuron-glia cultures, they induced the formation of large protein aggregates. After transduced into cell cultures, the CryAB^R120G^ mouse brain aggregate seeds triggered normal transduced CryAB proteins to become misfolded proteins and form large protein aggregates. Therefore, these results strongly suggest that peripherally misfolded proteins enhance I/R-induced neuroinflammation and brain injury possibly by the prion-like mechanism.

## Conclusion

In conclusion, we have demonstrated that following I/R, the misfolded CryAB^R120G^ proteins worsen neuroinflammation and brain injury in the mice. Furthermore, we have also demonstrated the role of exosomes in mediating CryAB^R120G^ proteotoxicity in both the mice and primary neuronal culture. Importantly, we provided strong evidence that the misfolded CryAB^R120G^ protein aggregate seeds can induce the normal CryAB protein into misfolded protein to form large protein aggregates in cell culture. These results suggest that the peripherally misfolded proteins disrupt brain function and exacerbate I/R-induced brain injury, which may represent an underappreciated mechanism underlying heart-brain crosstalk.

## Supplementary Information

**Additional file 1: Supplementary Fig. S1.** Cerebral blood flow does not differ between Ntg and CryAB^R120G^ mice before, during and after MCAO. Data are shown as mean ± SD. N = 3-4. **p < 0.01; $<0.001.

**Additional file 2: Supplementary Fig. S2.** Behavioral tests in the pre-MCAO mice. **a**. String agility test. **b**. Rotarod test. **c**. Y-maze test. Data are shown as mean ± SD, N = 7-8. *p < 0.05; n.s., no significant difference.

**Additional file 3: Supplementary Fig. S3.** Western blot analysis of the three indicated proinflammatory molecules in the peri-infarct cortex of Ntg and CryAB^R120G^ mouse brain at indicated time points following MCAO. The β-actin was used as a loading control.

**Additional file 4: Supplementary Fig. S4.** Thioflavin S staining of intact mouse brains absent of stroke insult. The brains from either Ntg or CryAB^R120G^ mice was sectioned and stained with Thioflavin S. Scale bar, 50 μm.

**Additional file 5: Supplementary Fig. S5.** Thioflavin S staining of Ntg or CryABR120G mouse brain following sham (upper panels) or 24 h of MCAO insult (lower panels). Scale bar, 50 μm.

**Additional file 6: Supplementary Fig. S6.** Abundance of large- (> 1 μm^2^, left), medium- (0.1–1 μm^2^, middle), and small-sized (< 0.1 μm^2^) protein aggregates in the two type of mouse brains. Each dot represents a protein aggregate with a specific surface area.

**Additional file 7: Supplementary Fig. S7.** Protein identification by mass spectrometry analysis. **a** Sample from the heart of CryAB^R120^G mice (Alpha-crystalline B chain mutant, positive control). **b** Sample from the brain of Ntg mice (Alpha-crystalline B WT, negative control).

**Additional file 8: Supplementary Fig. S8.** Thioflavin S staining of WT mouse brains following Ntg or CryAB^R120G^ exosome injection. WT mice were subjected to 1 h MCAO and after 1 h of reperfusion, the mice were intravenously injected either with Ntg or with CryAB^R120G^ exosomes before they were sacrificed at 24 h to assess protein aggregation in the brain by Thioflavin S staining. Scale bar, 30 μm.

## Data Availability

Data and materials supporting the findings of this manuscript are available from the corresponding author upon reasonable request.

## Abbreviations

CryAB^R120G^: Missense (R120G) mutant small heat shock protein, αB-crystallin
BBB: Blood-brain barrier
I/R: Ischemia and reperfusion
WT: Wild-type
Ntg: Non-transgenic
MCAO: Middle cerebral artery occlusion
MCA: Middle cerebral artery
ECA: External carotid artery
TTC: 2,3,5-Triphenyltetrazolium chloride
mNSS: Modified neurological severity score
GFAP: Glial fibrillary acidic protein
TNFα: Tumor necrosis factor-α
IL-1β: Interleukin-1β
IL-6: Interleukin-6
TCA: Trichloroacetic acid
NTA: Nanoparticle tracking analysis
SEM: Scanning electron microscopy

## References

[1] Chen B, Retzlaff M, Roos T, Frydman J (2011). Cellular strategies of protein quality control. Cold Spring Harb Perspect Biol.

[2] Leeman DS, Hebestreit K, Ruetz T, Webb AE, McKay A, Pollina EA, Dulken BW, Zhao X, Yeo RW, Ho TT (2018). Lysosome activation clears aggregates and enhances quiescent neural stem cell activation during aging. Science.

[3] Hu BR, Martone ME, Jones YZ, Liu CL (2000). Protein aggregation after transient cerebral ischemia. J Neurosci.

[4] Hochrainer K, Jackman K, Anrather J, Iadecola C (2012). Reperfusion rather than ischemia drives the formation of ubiquitin aggregates after middle cerebral artery occlusion. Stroke.

[5] Liu Y, Lu L, Hettinger CL, Dong G, Zhang D, Rezvani K, Wang X, Wang H (2014). Ubiquilin-1 protects cells from oxidative stress and ischemic stroke caused tissue injury in mice. J Neurosci.

[6] Liu Y, Qiao F, Wang H (2017). Enhanced proteostasis in post-ischemic stroke mouse brains by ubiquilin-1 promotes functional recovery. Cell Mol Neurobiol.

[7] Jucker M, Walker LC (2013). Self-propagation of pathogenic protein aggregates in neurodegenerative diseases. Nature.

[8] Li JY, Englund E, Holton JL, Soulet D, Hagell P, Lees AJ, Lashley T, Quinn NP, Rehncrona S, Bjorklund A (2008). Lewy bodies in grafted neurons in subjects with Parkinson's disease suggest host-to-graft disease propagation. Nat Med.

[9] Hansen C, Angot E, Bergstrom AL, Steiner JA, Pieri L, Paul G, Outeiro TF, Melki R, Kallunki P (2011). Fog K, et al: alpha-Synuclein propagates from mouse brain to grafted dopaminergic neurons and seeds aggregation in cultured human cells. J Clin Invest.

[10] He Z, Guo JL, McBride JD, Narasimhan S, Kim H, Changolkar L, Zhang B, Gathagan RJ, Yue C, Dengler C (2018). Amyloid-beta plaques enhance Alzheimer's brain tau-seeded pathologies by facilitating neuritic plaque tau aggregation. Nat Med.

[11] Ugalde CL, Finkelstein DI, Lawson VA, Hill AF (2016). Pathogenic mechanisms of prion protein, amyloid-beta and alpha-synuclein misfolding: the prion concept and neurotoxicity of protein oligomers. J Neurochem.

[12] Jeon I, Cicchetti F, Cisbani G, Lee S, Li E, Bae J, Lee N, Li L, Im W, Kim M (2016). Human-to-mouse prion-like propagation of mutant huntingtin protein. Acta Neuropathol.

[13] Zhang Z, Nie S, Chen L (2018). Targeting prion-like protein spreading in neurodegenerative diseases. Neural Regen Res.

[14] Wang X, Osinska H, Klevitsky R, Gerdes AM, Nieman M, Lorenz J, Hewett T, Robbins J (2001). Expression of R120G-alphaB-crystallin causes aberrant desmin and alphaB-crystallin aggregation and cardiomyopathy in mice. Circ Res.

[15] Cheema MU, Poulsen ET, Enghild JJ, Hoorn EJ, Fenton RA, Praetorius J (2013). Aldosterone and angiotensin II induce protein aggregation in renal proximal tubules. Physiol Rep.

[16] Mukherjee A, Soto C. Prion-like protein aggregates and type 2 diabetes. Cold Spring Harb Perspect Med. 2017;7. 10.1101/cshperspect.a024315 PMC5411686 28159831

[17] Chen Q, Liu JB, Horak KM, Zheng H, Kumarapeli AR, Li J, Li F, Gerdes AM, Wawrousek EF, Wang X (2005). Intrasarcoplasmic amyloidosis impairs proteolytic function of proteasomes in cardiomyocytes by compromising substrate uptake. Circ Res.

[18] Horwitz J (2003). Alpha-crystallin. Exp Eye Res.

[19] Lu L, Wang H (2012). Transient focal cerebral ischemia upregulates immunoproteasomal subunits. Cell Mol Neurobiol.

[20] Min JW, Liu Y, Wang D, Qiao F, Wang H (2018). The non-peptidic delta-opioid receptor agonist Tan-67 mediates neuroprotection post-ischemically and is associated with altered amyloid precursor protein expression, maturation and processing in mice. J Neurochem.

[21] Min JW, Lu L, Freeling JL, Martin DS, Wang H (2017). USP14 inhibitor attenuates cerebral ischemia/reperfusion-induced neuronal injury in mice. J Neurochem.

[22] Chen J, Sanberg PR, Li Y, Wang L, Lu M, Willing AE, Sanchez-Ramos J, Chopp M (2001). Intravenous administration of human umbilical cord blood reduces behavioral deficits after stroke in rats. Stroke.

[23] Adegoke OO, Qiao F, Liu Y, Longley K, Feng S, Wang H (2017). Overexpression of ubiquilin-1 alleviates Alzheimer's disease-caused cognitive and motor deficits and reduces amyloid-beta accumulation in mice. J Alzheimers Dis.

[24] Liu Y, Feng S, Subedi K, Wang H (2020). Attenuation of ischemic stroke-caused brain injury by a monoamine oxidase inhibitor involves improved proteostasis and reduced neuroinflammation. Mol Neurobiol.

[25] McMillin M, Frampton G, Grant S, Khan S, Diocares J, Petrescu A, Wyatt A, Kain J, Jefferson B, DeMorrow S (2017). Bile Acid-mediated sphingosine-1-phosphate receptor 2 signaling promotes neuroinflammation during hepatic encephalopathy in mice. Front Cell Neurosci.

[26] Xu N, Tang XH, Pan W, Xie ZM, Zhang GF, Ji MH, Yang JJ, Zhou MT, Zhou ZQ (2017). Spared nerve injury increases the expression of microglia m1 markers in the prefrontal cortex of rats and provokes depression-like behaviors. Front Neurosci.

[27] Norwood J, Franklin JM, Sharma D, D'Mello SR (2014). Histone deacetylase 3 is necessary for proper brain development. J Biol Chem.

[28] Liu Y, Min JW, Feng S, Subedi K, Qiao F, Mammenga E, Callegari E, Wang H (2020). Therapeutic role of a cysteine precursor, OTC, in ischemic stroke is mediated by improved proteostasis in mice. Transl Stroke Res.

[29] Baranyai T, Herczeg K, Onodi Z, Voszka I, Modos K, Marton N, Nagy G, Mager I, Wood MJ, El Andaloussi S (2015). Isolation of exosomes from blood plasma: qualitative and quantitative comparison of ultracentrifugation and size exclusion chromatography methods. PLoS One.

[30] Ge P, Luo Y, Liu CL, Hu B (2007). Protein aggregation and proteasome dysfunction after brain ischemia. Stroke.

[31] Liu Y, Qiao F, Wang H. Enhanced proteostasis in post-ischemic stroke mouse brains by ubiquilin-1 promotes functional recovery. Cell Mol Neurobiol. 2016. 10.1007/s10571-016-0451-3 PMC5462886 27928652

[32] Dong G, Callegari E, Gloeckner CJ, Ueffing M, Wang H (2012). Mass spectrometric identification of novel posttranslational modification sites in Huntingtin. Proteomics.

[33] Francisco NM, Hsu NJ, Keeton R, Randall P, Sebesho B, Allie N, Govender D, Quesniaux V, Ryffel B, Kellaway L, Jacobs M (2015). TNF-dependent regulation and activation of innate immune cells are essential for host protection against cerebral tuberculosis. J Neuroinflammation.

[34] Silva AA, Silva RR, Gibaldi D, Mariante RM, Dos Santos JB, Pereira IR, Moreira OC, Lannes-Vieira J (2017). Priming astrocytes with TNF enhances their susceptibility to Trypanosoma cruzi infection and creates a self-sustaining inflammatory milieu. J Neuroinflammation.

[35] Bras JP, Bravo J, Freitas J, Barbosa MA, Santos SG, Summavielle T, Almeida MI (2020). TNF-alpha-induced microglia activation requires miR-342: impact on NF-kB signaling and neurotoxicity. Cell Death Dis.

[36] Chen J, Cui C, Yang X, Xu J, Venkat P, Zacharek A, Yu P, Chopp M (2017). MiR-126 affects brain-heart interaction after cerebral ischemic stroke. Transl Stroke Res.

[37] Holmes BB, Furman JL, Mahan TE, Yamasaki TR, Mirbaha H, Eades WC, Belaygorod L, Cairns NJ, Holtzman DM, Diamond MI (2014). Proteopathic tau seeding predicts tauopathy in vivo. Proc Natl Acad Sci U S A.

[38] Suthahar N, Meijers WC, Sillje HHW, de Boer RA (2017). From inflammation to fibrosis-molecular and cellular mechanisms of myocardial tissue remodelling and perspectives on differential treatment opportunities. Curr Heart Fail Rep.

[39] Bonafede R, Turano E, Scambi I, Busato A, Bontempi P, Virla F, Schiaffino L, Marzola P, Bonetti B, Mariotti R (2020). ASC-Exosomes ameliorate the disease progression in SOD1(G93A) murine model underlining their potential therapeutic use in human ALS. Int J Mol Sci.

[40] Wren MC, Zhao J, Liu CC, Murray ME, Atagi Y, Davis MD, Fu Y, Okano HJ, Ogaki K, Strongosky AJ (2015). Frontotemporal dementia-associated N279K tau mutant disrupts subcellular vesicle trafficking and induces cellular stress in iPSC-derived neural stem cells. Mol Neurodegener.

[41] Ridder K, Keller S, Dams M, Rupp AK, Schlaudraff J, Del Turco D, Starmann J, Macas J, Karpova D, Devraj K (2014). Extracellular vesicle-mediated transfer of genetic information between the hematopoietic system and the brain in response to inflammation. PLoS Biol.

[42] van de Schootbrugge C, Schults EM, Bussink J, Span PN, Grenman R, Pruijn GJ, Kaanders JH, Boelens WC (2014). Effect of hypoxia on the expression of alphaB-crystallin in head and neck squamous cell carcinoma. BMC Cancer.

[43] Webster JM, Darling AL, Uversky VN, Blair LJ (2019). Small heat shock proteins, big impact on protein aggregation in neurodegenerative disease. Front Pharmacol.

[44] Arac A, Brownell SE, Rothbard JB, Chen C, Ko RM, Pereira MP, Albers GW, Steinman L, Steinberg GK (2011). Systemic augmentation of alphaB-crystallin provides therapeutic benefit twelve hours post-stroke onset via immune modulation. Proc Natl Acad Sci U S A.

[45] Yan H, Peng Y, Huang W, Gong L, Li L (2017). The protective effects of alphab-crystallin on ischemia-reperfusion injury in the rat retina. J Ophthalmol.

[46] Leak RK (2014). Heat shock proteins in neurodegenerative disorders and aging. J Cell Commun Signal.

[47] Brownell SE, Becker RA, Steinman L (2012). The protective and therapeutic function of small heat shock proteins in neurological diseases. Front Immunol.

[48] Hong Y, Zhao T, Li XJ, Li S (2017). Mutant huntingtin inhibits alphaB-crystallin expression and impairs exosome secretion from astrocytes. J Neurosci.

[49] Iwaki T, Wisniewski T, Iwaki A, Corbin E, Tomokane N, Tateishi J, Goldman JE (1992). Accumulation of alpha B-crystallin in central nervous system glia and neurons in pathologic conditions. Am J Pathol.

[50] Heneka MT, Kummer MP, Stutz A, Delekate A, Schwartz S, Vieira-Saecker A, Griep A, Axt D, Remus A, Tzeng TC (2013). NLRP3 is activated in Alzheimer's disease and contributes to pathology in APP/PS1 mice. Nature.

[51] Venegas C, Kumar S, Franklin BS, Dierkes T, Brinkschulte R, Tejera D, Vieira-Saecker A, Schwartz S, Santarelli F, Kummer MP (2017). Microglia-derived ASC specks cross-seed amyloid-beta in Alzheimer's disease. Nature.

[52] Stancu IC, Cremers N, Vanrusselt H, Couturier J, Vanoosthuyse A, Kessels S, Lodder C, Brone B, Huaux F, Octave JN (2019). Aggregated Tau activates NLRP3-ASC inflammasome exacerbating exogenously seeded and non-exogenously seeded Tau pathology in vivo. Acta Neuropathol.

[53] Deora V, Lee JD, Albornoz EA, McAlary L, Jagaraj CJ, Robertson AAB, Atkin JD, Cooper MA, Schroder K, Yerbury JJ (2020). The microglial NLRP3 inflammasome is activated by amyotrophic lateral sclerosis proteins. Glia.

[54] Li K, Li J, Zheng J, Qin S (2019). Reactive Astrocytes in Neurodegenerative Diseases. Aging Dis.

[55] Li Q, Haney MS (2020). The role of glia in protein aggregation. Neurobiol Dis.

[56] Nelson MLA, Hanna E, Hall S, Calvert M (2016). What makes stroke rehabilitation patients complex? Clinician perspectives and the role of discharge pressure. J Comorb.

[57] Fernando R, Drescher C, Nowotny K, Grune T, Castro JP (2019). Impaired proteostasis during skeletal muscle aging. Free Radic Biol Med.

[58] Shen Y, Yan B, Zhao Q, Wang Z, Wu J, Ren J, Wang W, Yu S, Sheng H, Crowley SD (2018). Aging is associated with impaired activation of protein homeostasis-related pathways after cardiac arrest in mice. J Am Heart Assoc.

