# Supplementary material for: Peripherally misfolded proteins exacerbate ischemic stroke-induced neuroinflammation and brain injury
Source: J Neuroinflammation. 2021 Jan 20;18:29. doi: 10.1186/s12974-021-02081-7 (PMC7818745; doi:10.1186/s12974-021-02081-7)
Supplement: Supplementary file 1 [file 12974_2021_2081_MOESM1_ESM.docx]

Supplementary Figure S1

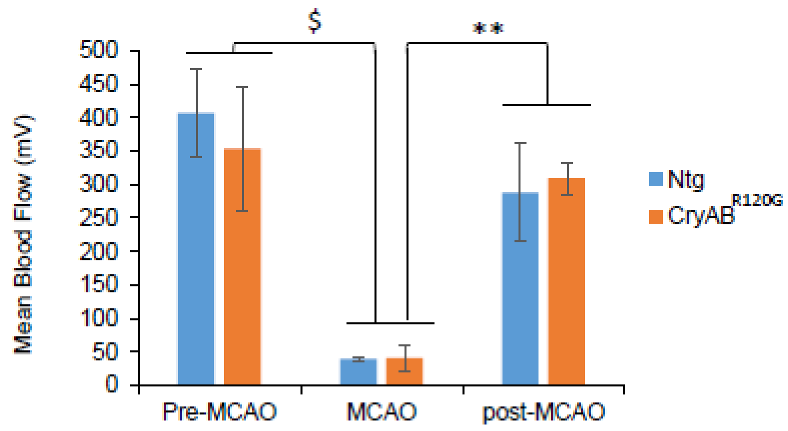

Supplementary Fig. S1: Cerebral blood flow does not differ between Ntg and CryAB^{R120G} mice before, during and after MCAO. Data are shown as mean ± SD. N = 3-4. **$p < 0.01$; *$<0.001.*
