# Supplementary material for: Peripherally misfolded proteins exacerbate ischemic stroke-induced neuroinflammation and brain injury
Source: J Neuroinflammation. 2021 Jan 20;18:29. doi: 10.1186/s12974-021-02081-7 (PMC7818745; doi:10.1186/s12974-021-02081-7)
Supplement: Supplementary file 2 [file 12974_2021_2081_MOESM2_ESM.docx]

Supplementary Figure S2

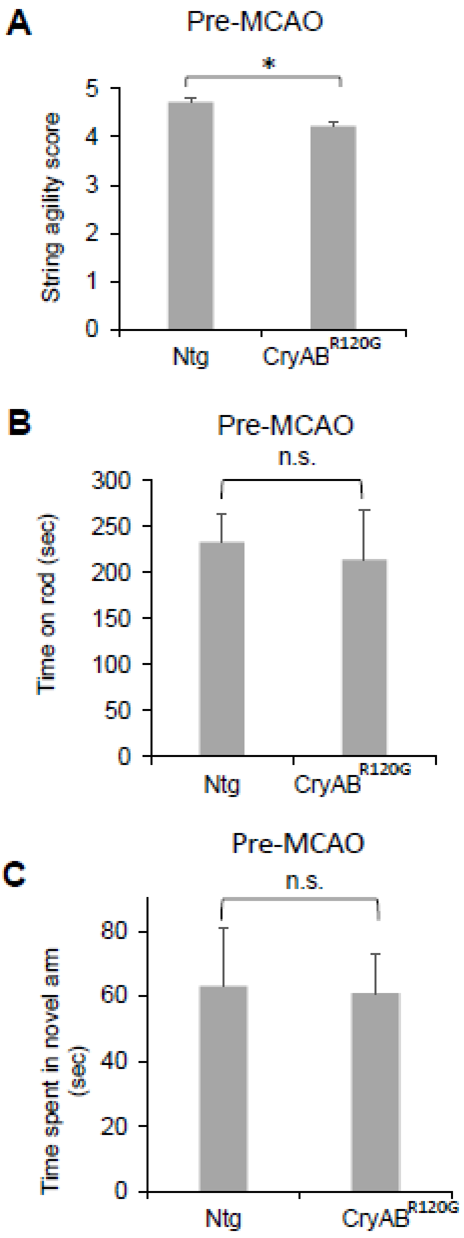

Supplementary Fig. S2: Behavioral tests in the pre-MCAO mice. A. String agility test. **B.** Rotarod test. **C.** Y-maze test. Data are shown as mean ± SD, N = 7-8. *$p < 0.05$; n.s., no significant difference.
