# Supplementary material for: Peripherally misfolded proteins exacerbate ischemic stroke-induced neuroinflammation and brain injury
Source: J Neuroinflammation. 2021 Jan 20;18:29. doi: 10.1186/s12974-021-02081-7 (PMC7818745; doi:10.1186/s12974-021-02081-7)
Supplement: Supplementary file 3 [file 12974_2021_2081_MOESM3_ESM.docx]

Supplementary Figure S3

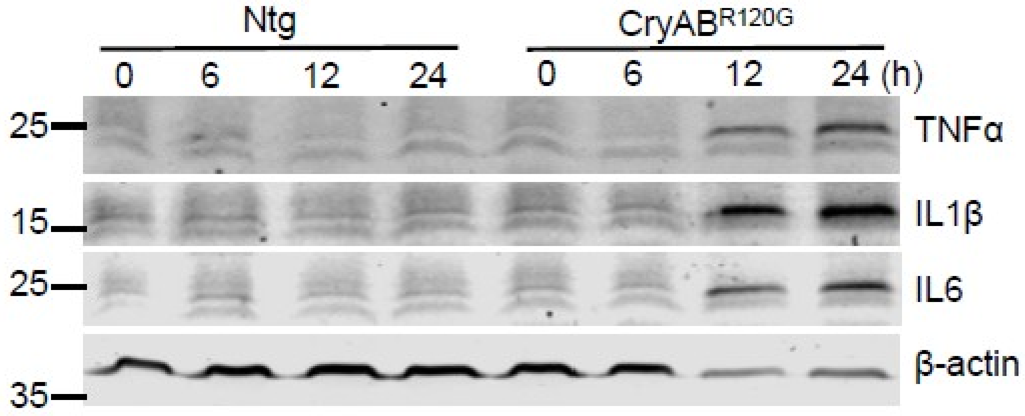

Supplementary Fig. S3: Western blot analysis of the three indicated pro-inflammatory molecules in the peri-infarct cortex of Ntg and CryAB^{R120G} mouse brain at indicated time points following MCAO. The β-actin was used as an loading control.
