# Supplementary material for: Peripherally misfolded proteins exacerbate ischemic stroke-induced neuroinflammation and brain injury
Source: J Neuroinflammation. 2021 Jan 20;18:29. doi: 10.1186/s12974-021-02081-7 (PMC7818745; doi:10.1186/s12974-021-02081-7)
Supplement: Supplementary file 4 [file 12974_2021_2081_MOESM4_ESM.docx]

Supplementary Figure S4

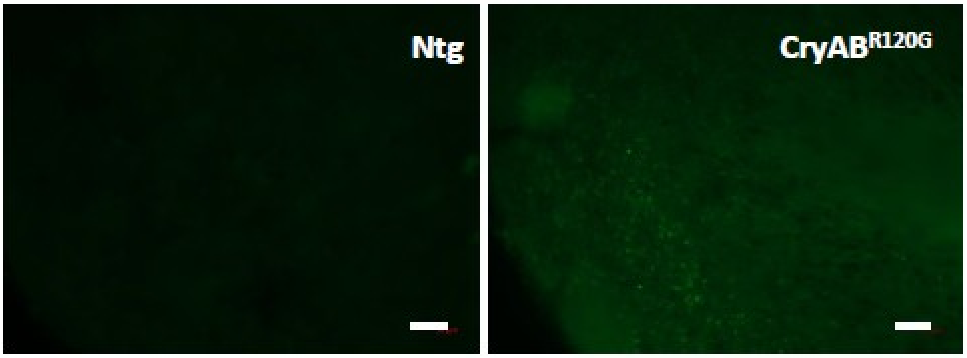

Supplementary Fig. S4: Thioflavin S staining of intact mouse brains absent of stroke insult. The brains from either Ntg or CryAB^{R120G} mice was sectioned and stained with Thioflavin S. Scale bar, 50 µm.
