# Supplementary material for: Peripherally misfolded proteins exacerbate ischemic stroke-induced neuroinflammation and brain injury
Source: J Neuroinflammation. 2021 Jan 20;18:29. doi: 10.1186/s12974-021-02081-7 (PMC7818745; doi:10.1186/s12974-021-02081-7)
Supplement: Supplementary file 5 [file 12974_2021_2081_MOESM5_ESM.docx]

Supplementary Figure S5

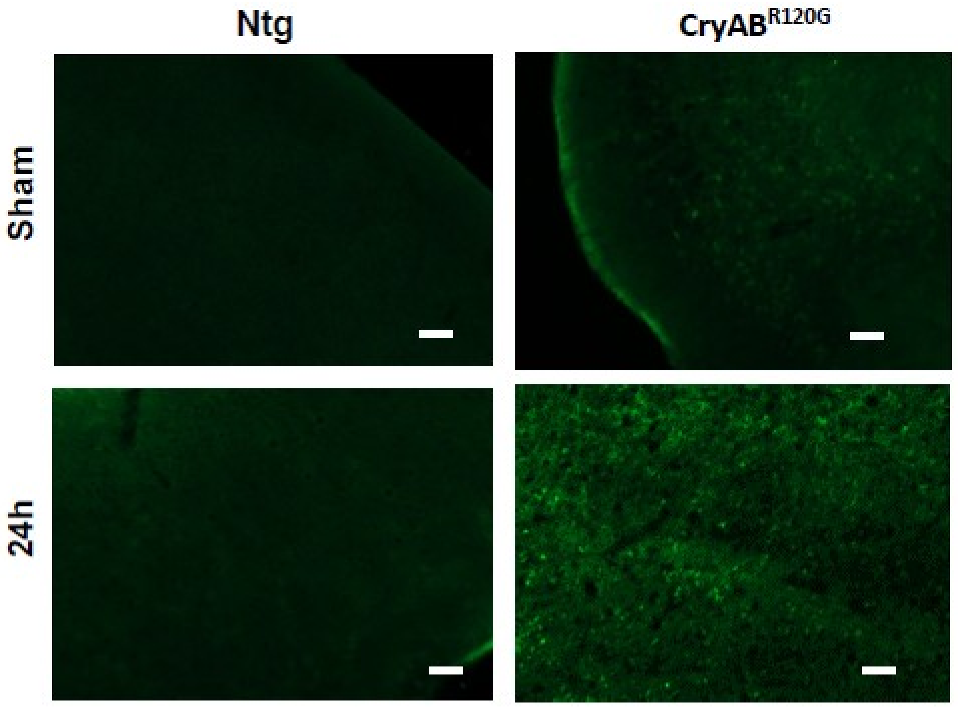

Supplementary Fig. S5: Thioflavin S staining of Ntg or CryAB^{R120G} mouse brain following sham (upper panels) or 24 h of MCAO insult (lower panels). Scale bar, 50 μm.
