# Supplementary material for: Peripherally misfolded proteins exacerbate ischemic stroke-induced neuroinflammation and brain injury
Source: J Neuroinflammation. 2021 Jan 20;18:29. doi: 10.1186/s12974-021-02081-7 (PMC7818745; doi:10.1186/s12974-021-02081-7)
Supplement: Supplementary file 6 [file 12974_2021_2081_MOESM6_ESM.docx]

Supplementary Figure S6

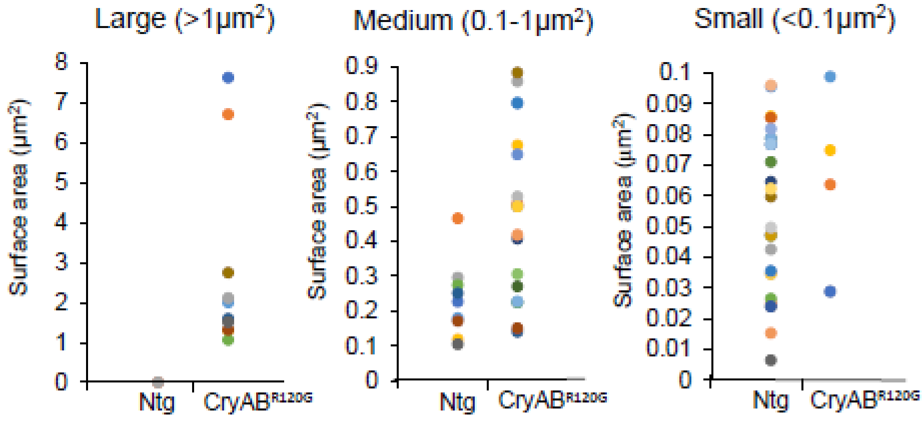

Supplementary Fig. S6: Abundance of large- (> 1 μm^2, left), medium- (0.1 - 1 μm^2, middle), and small-sized (< 0.1 μm^2) protein aggregates in the two type of mouse brains. Each dot represents a protein aggregate with a specific surface area.
