# Supplementary material for: Peripherally misfolded proteins exacerbate ischemic stroke-induced neuroinflammation and brain injury
Source: J Neuroinflammation. 2021 Jan 20;18:29. doi: 10.1186/s12974-021-02081-7 (PMC7818745; doi:10.1186/s12974-021-02081-7)
Supplement: Supplementary file 7 [file 12974_2021_2081_MOESM7_ESM.docx]

Supplementary Figure S7

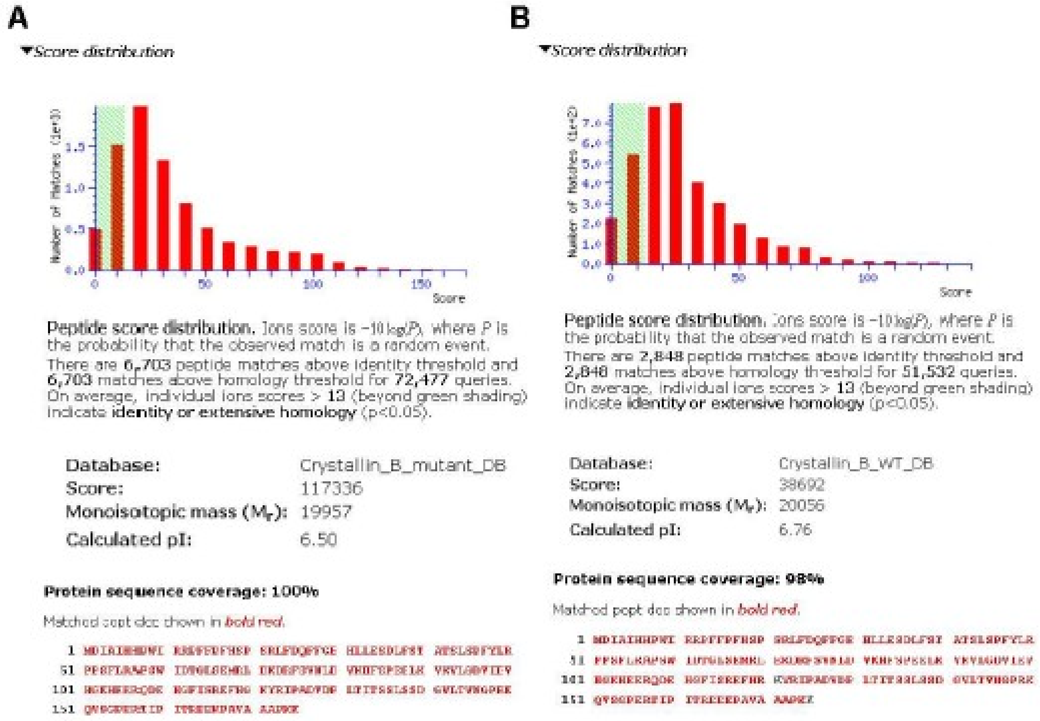

Supplementary Fig. S7: Protein identification by mass spectrometry analysis. (A) Sample from the heart of CryAB^{R120G} mice (Alpha-crystalline B chain mutant, positive control). (B) Sample from the brain of Ntg mice (Alpha-crystalline B WT, negative control).
