# Supplementary material for: Peripherally misfolded proteins exacerbate ischemic stroke-induced neuroinflammation and brain injury
Source: J Neuroinflammation. 2021 Jan 20;18:29. doi: 10.1186/s12974-021-02081-7 (PMC7818745; doi:10.1186/s12974-021-02081-7)
Supplement: Supplementary file 8 [file 12974_2021_2081_MOESM8_ESM.docx]

Supplementary Figure S8

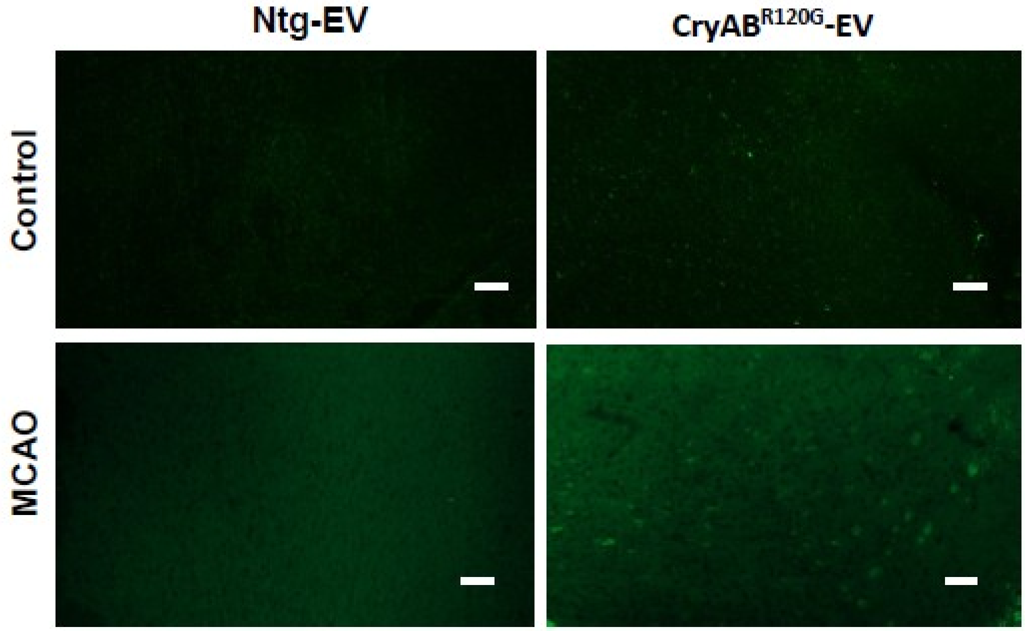

Supplementary Fig. S8: Thioflavin S staining of WT mouse brains following Ntg or CryAB^{R120G} exosome injection. WT mice were subjected to 1 h MCAO and after 1 h of reperfusion, the mice were intravenously injected either with Ntg or with CryAB^{R120G} exosomes before they were sacrificed at 24 h to assess protein aggregation in the brain by Thioflavin S staining. Scale bar, 30 µm.
